# Influence of herbal supplementation on nutrient digestibility, blood biomarkers, milk yield, and quality in tropical crossbred cows

**DOI:** 10.1371/journal.pone.0313419

**Published:** 2024-11-14

**Authors:** Md. Aliar Rahman, Md Rahat Ahmad Redoy, Abdullah Al Sufian Shuvo, Rakhi Chowdhury, Emran Hossain, Sheikh Mohammad Sayem, Md. Harun-ur- Rashid, Mohammad Al-Mamun

**Affiliations:** 1 Faculty of Animal Husbandry, Department of Animal Nutrition, Bangladesh Agricultural University, Mymensingh, Bangladesh; 2 Division of Administration and Common Service, Bangladesh Rice Research Institute, Joydebpur, Bangladesh; 3 Faculty of Agricultural Economics and Rural Sociology, Department of Agricultural and Applied Statistics, Bangladesh Agricultural University, Mymensingh, Bangladesh; 4 Faculty of Animal Husbandry, Department of Dairy Science, Bangladesh Agricultural University, Mymensingh, Bangladesh; Zagazig University Faculty of Agriculture, EGYPT

## Abstract

Herbal supplements containing diverse phytochemicals have been proven to improve digestibility, beneficial serum metabolites, milk yield, and quality in cows. However, the temperature sensitivity of phytochemicals in herbs complicates their practical use as livestock supplements. In response, this study aimed to assess how shade-dried plantain, lemongrass, and their combination influence cow performance, digestibility, serum and milk antioxidants, and milk fatty acids. Forty multiparous mid-lactating Holstein-tropical crossbred cows were randomly assigned to four groups (n = 10) for 63 days in a completely randomized design with covariates adjustment. The control group received a basal diet (14.9% crude protein, 11.0 MJ metabolizable energy/kg dry matter (DM)) without herbs. The herbs-supplemented groups received shade-dried powder (per cow/day) of either 100 g plantain (plantain), 100 g lemongrass (lemongrass), or 50 g each of plantain and lemongrass (combined), along with the basal diet. Compared with the control, the plantain or lemongrass group presented improved performance, i.e., DM consumption (3–4%), milk (10–11%) and its components yield (p ≤ 0.05). Similar to the control, the combined group had no effect on performance or digestibility, whereas the digestibility of all nutrients in the plantain or lemongrass group substantially increased. Herbs-supplementation reduced serum bad-cholesterol and urea-nitrogen while increasing good-cholesterol and albumin compared with the control (p < 0.01). Notably, 4–8% serum and 8–23% milk antioxidants were greater in the herbs-supplemented groups than the control, while the lemongrass had the optimum milk β-carotene. Substantially, herbs-supplementation resulted in a 6–10% higher unsaturated fatty acids (USFAs), whereas the combined group presented a 3.56-fold greater n-3 fatty acids than the control. Significantly, the plantain or lemongrass group presented better benefit‒cost‒ratio than the combined and control. Therefore, shade-dried herbs-supplementation improved the beneficial serum metabolites, serum and milk antioxidants, and milk USFAs in cows. Additionally, shade-dried plantain or lemongrass enhanced cow DM consumption, digestibility, milk yield, and profitability.

## 1. Introduction

In subtropical countries, e.g., Bangladesh, farmers often raise Holstein tropical crossbred cows and produce artificially inseminate pure Holstein (*Bos taurus*) with Zebu-type indigenous cows (*Bos indicus*) in an intensive farming system to meet the growing demand for milk. Nevertheless, the management practices of these cows often involve substandard housing, inadequate nutrition, and heat stress. These factors exacerbate their stress response, leading to an increase in free radical production and cell damage. As a result, milk yield and quality are negatively affected [1]. To address these issues, phytochemical-rich herbs, such as ginseng extract, grape pomace, moringa leaves, and various Chinese herbal preparations, have been used in the tropics to mitigate free radical production and improve serum and milk antioxidant levels, thus resulting in improved cow milk yield and quality [1,2]. Furthermore, these diverse benefits of herbs are attributed to their phytochemicals, such as phenols, flavonoids, terpenoids, and glycosides, which are influenced by their nature and dosage. The response of dairy cows to phytochemicals varies on the basis of the processing (microwave, oven, shade, sun, and freeze drying) and supplementation (extracts and powder in the diet or drinking water) methods of the herbs [3]. Freeze drying is considered the gold standard for preserving phytochemicals in herbal supplements. However, the unavailability of freeze dryers, along with the high cost and expertise required to operate this equipment, has prompted scientists to develop alternative strategies for conserving these phytochemicals in herbal supplements [3]. Shade-drying is a viable method for preserving phytochemicals economically and suppressing herb scents, which may affect cow milk [4,5].

Plantain (*Plantago lanceolata* L.) is a forage herb (forb) commonly used in climate-smart dairy cattle production [6,7]. It is rich in phytochemicals, including acteoside, aucubin, catalpol, tannin, and β-carotene [8]. These plantain phytochemicals exert anti-inflammatory, antioxidative, antiparasitic, and antimicrobial effects on animals, resulting in improved nutritional status and productivity [9]. As a component of fresh pasture, plantain has been shown to positively affect the rumen microbiome, improve milk yield, and reduce blood urea nitrogen in cows [10,11]. It contains acteoside, which is an influential antioxidant. Research has shown that supplementing donkeys with an acteoside (verbascoside)-based commercial additive (10 g/day) significantly reduces serum bad cholesterol and improves milk vitamin-A and desirable fatty acid contents (monounsaturated fatty acids and n-3 fatty acids) [12]. Additionally, fresh plantain (10 g dry matter (DM)/day/sheep) improves nutrient digestibility, growth, immunity, desirable meat fatty acids, serum total antioxidant capacity (TAC), superoxide dismutase (SOD), glutathione peroxidase (GPx), and catalase in sheep [13].

Lemongrass (*Cymbopogon citratus*) is a perennial grass grown in tropical and subtropical regions, including Bangladesh. It is used as a feed supplement for livestock, especially ruminants [14]. It acts as an antioxidant, antibacterial, anti-inflammatory, and antimicrobial agent because of its citral, β-myrcene, limonene, saponin, flavonoid, and tannin contents [14,15]. These properties enhance the beneficial rumen microbiome, blood metabolites, milk and component yields, and unsaturated fatty acids (USFAs) contents of ewes [15,16]. Lemongrass powder (100 g/day/cattle) improves nutrient utilization and microbial protein synthesis in beef cattle by positively altering the rumen microbiome [4]. However, it has inconsistent effects on nutrient digestibility, blood cholesterol, and urea nitrogen in Holstein steers [17] and finisher lambs [18]. Moreover, the main phytochemical in lemongrass, citral, aids in the synthesis of vitamin-A. This vitamin-A decreases the production of free radicals and increases the concentrations of serum TAC, SOD, GPx, and catalase in dairy cows [19].

In addition to their therapeutic properties, both herbs are rich in USFAs and various phytochemicals, such as quercetin and gallic acid, which can be transported from the blood into milk via feed [20]. Moreover, the expectation that shade-dried herbs’ phytochemicals will be transmitted to the mammary gland and excreted through milk is based on the metabolic pathways of these compounds. Phytochemicals such as acteoside in shade-dried plantain and citral in shade-dried lemongrass can be absorbed into the bloodstream, where they exert systemic effects, including potential benefits to various organs and tissues. Given that the mammary gland is highly vascularized and active in lactating dairy cows, various phytochemicals and beneficial fatty acids may reach the glands and be incorporated into milk. Hence, this study aimed to evaluate the effects of shade-dried plantain, lemongrass, or their combination on DM intake; milk yield; nutrient digestibility; serum lipid, protein, and serum and milk antioxidant (TAC, SOD, GPx, and catalase) contents; and milk quality (vitamin and fatty acid profiles) in Holstein tropical crossbred cows.

## 2. Materials and methods

### 2.1. Ethics statement

Cow handling and sampling were performed in accordance with the rules of the Animal Welfare and Experimentation Ethics Committee (AWEEC) of Bangladesh Agricultural University (BAU; AWEEC/BAU/2021/60).

### 2.2. Experimental design, animals, management, and feeding

This research was conducted on a dairy farm (location 24°71′22.7″N, 90°42′80.9″E) adjacent to BAU, Bangladesh. Forty multiparous mid-lactating Holstein-tropical crossbred cows (50% Holstein × 50% Zebu-type indigenous cows) were arbitrarily allotted to one of four dietary groups (n = 10) in a completely randomized design with three covariates adjustments. The study was conducted over a 56 days collection period following a 7 days adaptation period for the basal diet and herbs. The covariates were days in milk (DIM), parity number (PN), and body weight (BW). At the beginning of the trial, the initial milk yield of the cows averaged 8.35 ± 1.28 kg/day, with DIM 102 ± 35, PN 3.0 ± 1.0, and BW 432 ± 38 kg.

The cows were housed in a face-out arrangement in a tie-stall, ensuring a similar environment (temperature 27.0 ± 3.0°C, relative humidity 70.0 ± 3.0%). Each cow was kept in a separate stall providing ideal space (40 square feet), and equipped with a deep manger (length 2.5 feet, width 2.5 feet, height 1.5 feet front and 3.0 feet back) to ensure feed consumption. The cows were hand-milked twice daily at 0700 and 1600 h. Cows had *ad libitum* access to clean water via a deep water tube (length 2.0 feet, width 2.5 feet, height 1.5 feet front and 3.0 feet back) throughout the day. The cows and stalls were cleaned twice daily. Just before the 14^th^ day of the trial, the cows were administered Invermac Plus (G-live, Bangladesh) at a dose of 2.0 ml/100 kg BW.

The cows received a basal diet with 14.9% crude protein (CP) and 11.0 MJ metabolizable energy/kg DM without any herbal supplements, and were considered as the control group. The herbs-supplement groups were given the basal diet plus herbs (per cow/day), which included either 100 g of shade-dried plantain powder, 100 g of shade-dried lemongrass powder, or a combination of 50 g each of shade-dried plantain and lemongrass powder. These groups were designated as the plantain, lemongrass, and combined groups, respectively. The basal diet consisted of 51% concentrates (wheat bran, mustard oil cake, broken rice, ready-mix feed, di-calcium phosphate, and salt) and 49% German grass (*Echinochloa polystachya*; Table 1). The concentrates and grass were offered to all cows in similar portions twice daily, just before (0630 and 1530 h) and after milking (0900 and 1800 h), respectively. Each cow in all the groups received a daily supplement of 7.35 kg of concentrate. The grass was cut and chopped twice daily and offered to each cow on a fresh and *ad libitum* basis. To supply *ad libitum* grass, daily grass intake was calculated, and 1.10 times the amount of grass consumed the next day was supplied to ensure that the amount of grass consumed was 10% greater than its voluntary intake. The herbal supplements were supplied daily before morning milking and inspection confirmed their consumption. The ingredients and nutrient composition of the basal diet, and herbal supplements are detailed in Table 1.

**Table 1:** Ingredient composition and nutritional attributes of basal diet and supplemented herbs.

Ingredients composition of basal diet		Nutrient composition of the ingredients in basal diet				
Ingredients	(g/kg DM)	Dry matter (as fed basis)	(g/kg DM)			
			CP	CF	EE	Ash
German grass	488.8	189.1	114.5	347.1	297.0	103.0
Wheat bran	254.4	896.9	207.4	69.9	31.7	45.9
Mustard oil cake	88.5	920.8	347.8	111.3	116.7	61.0
Broken rice	73.6	897.5	87.2	10.4	16.9	6.1
Ready-mix feed	72.4	895.2	192.8	67.3	39.7	123.5
Common salt	16.5	981.7	-	-	-	996.4
Di-calcium phosphate	5.9	969.4	-	-	-	903.2
***Nutrient composition of basal diet and supplemented herbs (g/kg DM)***						
		**Basal diet**	**Plantain herb**		**Lemongrass**	
Dry matter (DM)		312.1	896.7		915.2	
Organic matter		903.3	845.5		911.4	
Crude protein (CP)		149.3	150.7		53.7	
Neutral detergent fiber		383.3	360.1		631.4	
Acid detergent fiber		223.3	243.8		411.4	
Ether extract (EE)		47.1	29.6		51.5	
Ash		96.7	154.5		88.6	
Calcium		6.61	1.13		0.40	
Phosphorus		4.63	2.50		0.91	
Metabolizable energy (MJ/kg)		11.00	9.02		9.65	
***Antioxidant attributes***						
DPPH (%)		-	27.43		15.27	
TPC (mg GAE/g dry sample)		-	24.67		19.83	
TFC (mg QE/g dry sample)		-	5.29		10.51	
***Fatty acid profile (%)***						
Saturated fatty acids		23.40*	20.40		25.67	
Unsaturated fatty acids		76.60*	79.60		74.33	
Monounsaturated fatty acids		16.27*	92.88		87.52	
Polyunsaturated fatty acids		60.33*	7.12		12.48	
n-6 fatty acids		49.06*	19.36		15.67	
n-3 fatty acids		11.27*	46.07		57.89	

DPPH = 2, 2‒diphenyl‒1‒picryl hydrazyl; TPC = total phenolic contents; GAE = gallic acid equivalent; TFC = total flavonoid contents; QE = quercetin equivalent.

*Calculated value based on the fatty acid profiles of ingredients used in the basal diet, according to the NASEM 2021.

The grasslands Lancelot plantain (*Plantago lanceolata* L.) and West Indian lemongrass (*Cymbopogon citratus* DC. Staf) were cultivated in a well-tilled sandy loam highland plot with adequate drainage, irrigation, weeding, and optimal environmental conditions at Shahjalal Animal Nutrition Field Laboratory, BAU. The plantain was harvested at 65 days, whereas the lemongrass was harvested at 60 days [13]. Both herbs were dehydrated in a shaded area for 4‒5 days at 27.0 ± 3.0°C with proper wilting and a pedestal fan (1240 rotations per minute). The dried herbs were ground into 1 mm particles via a locally made feed grinder and placed in airtight bags. This herb bag was then kept at room temperature to prevent nutritional and physical changes due to growth of mold and fungus.

### 2.3. Dry matter intake, apparent nutrient digestibility, samples collection, and preparation

Daily German grass intake was determined by subtracting refusal grass from the grass offered to each cow, whereas concentrate intake was recorded. To minimize moisture variations in the grass and optimize accurate DM intake, weekly grass and monthly concentrate and herb powder samples were collected and oven-dried at 105°C for 24 h to determine their DM [21]. The total DM intake for each cow was subsequently determined from the grass, concentrate, and herb powder DM records. Additionally, to determine the apparent nutrient digestibility, along with recording daily feed intake, a total collection of feces was conducted for the last 7 days of the study. To avoid contamination of feces with urine, the total feces of each cow was collected from each stall every 3 h over a 24 h period through continuous monitoring and stored in covered containers. The daily fecal output for each cow was weighed to determine the total fecal output. After being weighed, a 50 g fresh sample was stored at −20°C to make composite samples for further proximate analysis, while 10 g fresh fecal and 10 g fresh grass samples were collected for DM determination. Both samples from each cow were subsequently dried daily at 105°C for 24 h in a micro-oven to determine the DM content [21]. This process was repeated daily for 7 days. After 7 days, a 350 g composite sample was made for each cow from the daily samples after thawing. These composite samples were oven-dried (70°C) and ground (1 mm) using a locally made grinder for proximate analysis. The apparent nutrient digestibility was subsequently evaluated by calculating the ratio of the difference between the intake and fecal nutrient outgo to the intake nutrient, and expressed as a percentage. In addition, the grass was oven dried (70°C for 48 h) and processed, and the basal diet ingredients were weighed accurately. The basal diet was then ground into a 1 mm powder via a locally made electric grinder. The basal diet and previously ground shade-dried herbal powder were stored at −20°C until analysis.

The daily milk yield was measured and recorded separately as the morning and afternoon milk yield. A milk sample of 60 ml from the morning and 40 ml from the afternoon (100 ml) was collected into two separate falcon tubes (50 ml each) on days 14, 28, 42, and 56 after the adaptation period for the determination of milk antioxidant levels. Like in previous samples, milk was collected four times, mixed uniformly, dried via a freeze dryer (SL no. 60000295, DC-401, Yamato, Japan), and stored at −20°C for β-carotene analysis. On the final day of the trial, a 50 ml sample of milk (morning: afternoon = 3: 2) was collected and preserved at −20°C for analysis of the fatty acid profiles. After 4 h of feeding, 10 ml blood sample was drawn from the jugular vein of dairy cows on days 14, 28, 42, and 56 using syringes equipped with 19 g needles. The blood sample was placed in a 15 ml sterile falcon tube and coagulated for 30 minutes. The samples were then centrifuged (Z 306, Hermle, Germany) at 3,000 × g for 15 minutes to harvest the serum, which was subsequently stored in Eppendorf tubes at −20°C pending analysis.

### 2.4. Diet, feces, and herbs samples analysis

The proximate components of the prepared basal diet and its ingredients, feces, and herbs samples were analyzed according to the AOAC [21]. The organic matter (OM) content of all the samples was obtained by subtracting the ash percentage from 100. The neutral detergent fiber (NDF) and acid detergent fiber (ADF) were measured according to the method of Goering and Van Soest [22]. For the determination of calcium (Ca) and phosphorus (P), the basal diet and herbs samples were digested with 10 ml of 6 mol/l HCl after ash determination at 550°C for 5 h. The digested sample was gradually raised at 160–170°C until fumes of HClO_4_ appeared via a locally made digester. The digested sample volume was adjusted to 25 ml by adding distilled water and was used for the determination of Ca and P via an UV‒visible spectrophotometer (T60; PG Instruments, UK). The antioxidant capacity of the herbs was measured via 2, 2‒diphenyl‒1‒picrylhydrazyl (DPPH) radical via a T60 UV‒visible spectrophotometer according to previous method [23]. The total phenolic content as a gallic acid equivalent (GAE; Kat No. 91215, Sigma Aldrich, Germany) and the total flavonoid content as a quercetin equivalent (QE; Kat No. Q4951, Sigma Aldrich, Germany) were determined in both herbs on the basis of the Folin-Ciocalteau and aluminum chloride colorimetric assays, respectively [24].

### 2.5. Serum lipid and protein indices analysis

After thawing, the lipid and protein profiles of the serum samples were analyzed on a bioanalyzer (Urit-810, China). The levels of high-density lipoprotein cholesterol (HDL-C), triglyceride, and total cholesterol (TC) in the serum were measured with enzymatic kits (Human Company, USA) in accordance with the manufacturer’s instructions. Low-density lipoprotein cholesterol (LDL-C) and very low-density lipoprotein cholesterol (VLDL-C) concentrations were determined in the serum via the following formula: LDL-C (mg/dl) = serum TC ─ HDL-C ─ (triglyceride/5), and VLDL-C (mg/dl) = serum triglyceride/5. Using enzymatic kits (Human Company, USA), total protein, urea, and albumin in the serum were quantified with an Urit-810 bioanalyzer, and globulin levels were obtained by subtracting albumin from total protein. In addition, blood urea nitrogen (BUN) was computed by dividing the urea value by 2.14, according to the manufacturer’s instructions.

### 2.6. Serum and milk antioxidants enzymes analysis

A 20 μl serum or milk sample with a protein mask was placed in a well plate to determine the serum or milk TAC, respectively. Then, the volume was brought to 100 μl with deionized water. In addition, 0, 4, 8, 12, 16, or 20 μl of 1 mM Trolox standard solution was added to the well plates. The volume was then increased to 100 μl using deionized water. Each sample and standard well received on addition of 100 μl of cupric cation solution. The well plate was shaken horizontally and kept at normal temperature without light for 90 minutes. Afterward, the wells were read at 570 nm via enzyme-linked immunosorbent assay (ELISA; EL-10A; Biobase, China), as provided in the kit’s instructions (Kat no. MAK187-1KT; Sigma Aldrich, Germany). A 20 μl serum or milk sample was added to 200 μl tetrazolium salt, 20 μl buffer, or 20 μl enzyme mixture to measure the serum or milk SOD, respectively. The mixture was maintained at 37°C for 20 minutes. The reading was then taken at 450 nm according to the SOD kit’s instructions (Kat no. K-335, BioVision, USA). The concentration of GPx in the serum or milk was measured by adding 20 μl of either the serum or milk sample, with similar amounts (50 μl) of assay buffer, co-substrate mixture, and nicotinamide adenine dinucleotide phosphate hydrogen, respectively. The absorption was subsequently measured at a wavelength of 356 nm, as suggested by the GPx kit (Kat no. K773, BioVision, USA). To measure the serum or milk catalase concentration, an H_2_O_2_ standard curve was prepared with different amounts of 1 mM H_2_O_2_ solution, catalase buffer, and stop solution. The solution used for catalase determination was prepared from either serum or milk sample (30 μl), a positive control, a high control, a stop solution, 1 mM H_2_O_2_, and developer mix. The mixture was then maintained at 25°C for 10 minutes. Finally, the absorbance was determined at 570 nm in a plate reader in accordance with the catalase kit’s instructions (Kat no. K762, BioVision, USA). In addition, the antioxidant capacity of milk was measured via the DPPH test [23]. For this test, 2 ml of 0.2 mmol/l DPPH solution was added to a test tube, mixed with a 100 μl milk sample and incubated at room temperature for 30 minutes. Then, 1 ml of chloroform was added, and the mixture was centrifuged at 3000 × g for 5 minutes. A 100 mmol/l DPPH solution was prepared in methanol and used as the control. A T60 UV‒visible spectrophotometer was used to measure the absorbance of the clear solution at 517 nm. The following equation was used: scavenging activity percent = [(absorbance of the control ─ absorbance of the sample)/absorbance of the control] × 100. The percentage inhibition of the DPPH free radical (scavenging percentage) was determined on the basis of the reading of the control solution.

### 2.7. Milk β-carotene, composition, and fatty acid and herb fatty acid profiles analysis

In addition, milk β-carotene was extracted from freeze-dried milk samples via hexane extraction in a brown paper bag at room temperature [25] via an UV‒visible spectrophotometer (DU® 730, Beckman Coulter). Approximately 100 ml fresh milk samples were taken in the morning from each cow to determine the raw milk composition at 14, 28, 42, and 56 days. After milk sampling, the samples were analyzed within 2 h via the MilkoScan SLP-60 to quantify milk total solids (TS), fat, solids-not-fat (SNF), lactose, and minerals. A 0.5 ml milk sample was analyzed to measure protein levels [21]. The energy-corrected (ECM) and 4% fat-corrected (FCM) milk values were calculated by adopting the definitions of ALP [26] and Tyrrell and Reid [27], respectively. The fatty acid profiles of both herbs and milk samples were determined via a gas chromatograph equipped with a flame ionization detector (GC-FID; Model 14B SHIMADZU) operated with Class GC-10 software (version 2.00). The GC-FID was set with a capillary column (Restek®, serial No. 1324355; USA) with dimensions of 15 m in length and 2.25 mm in internal diameter.

### 2.8. Cost‒benefit analysis

Throughout the trial, with the exception of feed costs, all costs were similar across the dietary groups. The price of fresh herbs was 0.091$/kg, and the processing cost was 0.027$/kg. After adjusting to the DM content of fresh plantain (14.97%) and lemongrass (27.03%), the price/kg of DM was 0.788$ for plantain and 0.437$ for lemongrass. The prices (kg/DM) for the concentrate mixture and German grass were 0.376$ and 0.238$, respectively. The selling price of fresh milk was 0.636$/kg for all dietary groups without considering the level of antioxidants in milk. The benefit‒cost‒ratio (BCR) on total feed cost (TFC) was calculated as the ratio of benefits to TFC.

### 2.9. Data analysis

Excel and SPSS 22 were used to record and analyze the raw data, respectively. Each cow had a separate pen and was an experimental unit. The initial milk yield of the four dietary groups was analyzed via one-way ANOVA, considering DIM, PN, and BW as covariates. The analysis revealed a homogenous milk yield across the dietary groups (p = 0.92). At the start of the trial, BW (p = 0.73), DIM (p = 0.22), and PN (p = 0.74) were also similar among the groups. Samples from each cow for all parameters except nutrient digestibility and milk fatty acid profiles were collected four times (14, 28, 42, and 56 days). Each sample was analyzed in triplicate, whereas three fecal and two milk samples from each cow were collected and analyzed for nutrient digestibility and fatty acids determination, respectively. However, on the 55^th^ day, two cows in the combined group were affected by subclinical mastitis. Milk from cows with subclinical mastitis was greater in free fatty acids, polyunsaturated fatty acids (PUFAs), and short-chain fatty acids but lower in long-chain fatty acids [28]. This may be the reason that a mixture of plantain and lemongrass fails to affect milk fatty acids. Moreover, 20 milk samples from 10 cows in each group except the combined group (8 cows = 16 milk samples) were analyzed to measure the fatty acid profiles. One-way repeated measures ANCOVA were used in this research to assess the significant factors associated with the response variables. The subsequent model illustrates the repeated measures ANCOVA for all response variables, excluding nutrient digestibility, milk fatty acid profiles, and cost‒benefit analysis.

Yij=αi+Di+Tj+DiTj+β1DIM+β2PN+β3BW+εij;i,j=1,2,3,4.

Y defines the response variable, αi signifies the average value of the output accounting for the random effects attributed to individual cows, as well as D, T, and DT represent diet, time, and the interaction between diet and time, respectively, which are regarded as fixed effects. Additionally, DIM, PN, and BW indicate days in milking, parity number, and body weight, respectively, which are treated as covariates.

In addition, the nutrient digestibility, milk fatty acid profiles, and cost‒benefit of dairy cows were analyzed via one-way ANOVA. During the cost‒benefit analysis of dairy cows, the prices of feed ingredients and milk, along with the analyzed values of DM intake through grass, concentrate, and herbs, as well as milk yield (Table 2), were taken into account to minimize errors related to cows with DM intake and lower milk yield. Significant differences among the dietary means were assessed through post hoc (LSD) methods at 0.01 ≤ p value ≤ 0.05.

**Table 2:** Effects of herbal supplementation on milk production performances in tropical crossbred cows.

Variables	Control	Herbs-supplemented groups			SEM	p-value*		
		Plantain	Lemongrass	Combined		D	T	D × T
**Dry matter intake (DMI; kg/day)**								
Total	13.05^b^	13.59^a^	13.46^a^	13.27^ab^	0.04	0.01	0.01	0.95
Concentrate	6.65	6.65	6.65	6.65	0.00	1.00	1.00	1.00
German grass	6.40^b^	6.85^a^	6.72^a^	6.54^b^	0.03	0.01	0.01	0.93
Herbs	0.00^b^	0.09^a^	0.09^a^	0.09^a^	0.00	<0.01	1.00	1.00
**Milk performance (kg/day)**								
Milk yield	7.96^b^	8.83^a^	8.73^a^	8.20^ab^	0.12	0.05	0.22	1.00
ECM	7.57^b^	7.99^ab^	8.39^a^	7.72^b^	0.13	0.07	0.01	0.86
4% FCM	7.90	8.28	8.51	7.84	0.11	0.14	0.01	0.89
FE, DMI/ECM	1.77	1.71	1.63	1.76	0.02	0.13	0.11	0.93
**Milk components yield (g/day)**								
TS	958^b^	1038^ab^	1071^a^	1001^ab^	14.0	0.05	0.01	0.91
SNF	647^b^	722^a^	736^a^	697^ab^	10.3	0.02	0.07	0.90
Lactose	326^b^	364^a^	365^a^	345^ab^	5.21	0.04	0.06	0.90
Protein	263^b^	294^a^	309^a^	287^ab^	4.79	0.02	0.34	0.63
Fat	314^ab^	317^ab^	335^a^	304^b^	4.67	0.07	0.01	0.69

Control group = basal diet consists of German grass and concentrate with 14.9% CP and 11.0 MJ ME/kg DM without herbs; Plantain group = basal diet + 100 g shade-dried plantain powder/cow per day; Lemongrass group = basal diet + 100 g shade-dried lemongrass powder/cow per day; Combined group = basal diet + 50 g shade-dried plantain and 50 g shade-dried lemongrass powder/cow per day; SEM = standard error of mean.

*Probability of treatment effects: D = effects of experimental diets; T = effect of time; D × T = interaction between experimental diets and time; ^ab^Means within a row, denoted by distinct superscripts indicate significant differences (p ≤ 0.05) for the experimental diets.

ECM = energy-corrected milk; FCM = fat-corrected milk; FE = feed efficiency; TS = total solids; SNF = solids-not-fat.

## 3. Results

### 3.1. Production performance

Compared with those of the control group (p ≤ 0.05), improved DM intake (3–4%), milk yield (10–11%), and ECM (6‒11%) were observed in the cows given individually plantain or lemongrass, whereas FCM (**p** *=* 0.14) and feed efficiency (p *=* **0**.13) were similar among all the groups (Table 2). In addition, the cows in all the groups had similar concentrate intakes (p > 0.05). However, in comparison with those in the control and combined groups, the grass intake in the individual plantain or lemongrass group was greater (p < 0.05). Furthermore, the individual plantain or lemongrass group presented greater milk yields of TS, SNF, fat, lactose, and protein than did the control group (p *≤* 0.05). In addition, the combined group tended toward better DM intake, milk yield, ECM, and milk yields of TS, SNF, lactose, and protein than did the control (**p** *>* 0.05).

### 3.2. Apparent nutrient digestibility

Compared with the control, the cows offered plantain or lemongrass individually, resulted a 3–4% increase in DM and OM digestibility (p < 0.01), whereas the DM and OM digestibility of the combined group was comparable to that of the control group (p > 0.05; Table 3). However, the herbal supplementation group had lower CP digestibility than did those in the control group, while the plantain group showing the lowest value (p < 0.01). In addition, the plantain or lemongrass group had overall 5–7% greater crude fiber (CF) and NDF digestibility than did the control group, whereas the combined group was similar to the control group (p > 0.05). Additionally, compared with the control group, the herbal supplementation groups presented 3–9% greater ADF digestibility (p < 0.01), and almost 2–3% better ether extract (EE) digestibility was observed in the herbal supplementation groups than in the control group (p *=* 0.02).

**Table 3:** Effects of herbal supplementation on apparent nutrient digestibility in tropical crossbred cows.

Apparent nutrient digestibility (%)	Control	Herbs-supplemented groups			SEM	p-value
		Plantain	Lemongrass	Combined		
Dry matter	66.86^b^	69.70^a^	69.11^a^	67.95^ab^	0.30	<0.01
Organic matter	68.49^b^	70.64^a^	70.60^a^	68.86^b^	0.28	<0.01
Crude protein	71.85^a^	68.63^c^	71.42^a^	69.51^b^	0.32	<0.01
Crude fiber	63.25^b^	68.04^a^	66.92^a^	64.69^b^	0.50	<0.01
Neutral detergent fiber	61.34^b^	65.25^a^	64.87^a^	62.74^b^	0.44	<0.01
Acid detergent fiber	51.57^c^	55.45^a^	56.07^a^	52.89^b^	0.47	<0.01
Ether extract	79.20^b^	81.44^a^	81.85^a^	80.71^a^	0.31	0.02

Control group = basal diet consists of German grass and concentrate with 14.9% CP and 11.0 MJ ME/kg DM without herbs; Plantain group = basal diet + 100 g shade-dried plantain powder/cow per day; Lemongrass group = basal diet + 100 g shade-dried lemongrass powder/cow per day; Combined group = basal diet + 50 g shade-dried plantain and 50 g shade-dried lemongrass powder/cow per day; SEM = standard error of mean.

^a–c^Means within a row, denoted by distinct superscripts indicate significant (p ≤ 0.05).

### 3.3. Blood biomarkers

Herbal supplementation reduced the serum triglyceride and TC levels by 8–17% and 21–30%, respectively, compared with those of the control, with the lemongrass or plantain group showing the lowest triglyceride and TC levels (**p** *<* 0.01; Table 4). Nevertheless, herbal supplementation had inconsistent results in terms of the total protein level in the serum; however, it caused substantial increases (7–13%) in the serum albumin concentration and decreases (5–13%) in the serum globulin concentration (**p** *≤* 0.01), whereas the combined group had a level of globulin comparable to that of the control group (**p** > 0.05). Moreover, compared with the control group, the plantain group presented a lower serum BUN level (─19%; **p** *<* 0.01).

**Table 4:** Effects of herbal supplementation on serum lipid and protein indices in tropical crossbred cows.

Variables	Control	Herbs-supplemented groups			SEM	p-value*		
		Plantain	Lemongrass	Combined		D	T	D × T
**Serum lipid profile (mg/dl)**								
Triglyceride	24.62^a^	22.47^b^	20.84^c^	21.48^c^	0.12	<0.01	0.02	0.63
TC	172.18^a^	121.35^c^	137.01^b^	135.55^b^	0.81	<0.01	<0.01	0.02
HDL-C	66.29^b^	74.54^a^	67.48^b^	66.80^b^	0.36	<0.01	0.07	0.01
LDL-C	100.97^a^	42.31^c^	65.36^b^	64.46^b^	0.80	<0.01	<0.01	<0.01
VLDL-C	4.92^a^	4.17^c^	4.30^b^	4.49^b^	0.03	<0.01	0.02	0.63
**Serum protein indices (mg/dl)**								
Total protein	6.69^ab^	6.75^a^	6.57^b^	6.86^a^	0.03	0.01	<0.01	<0.01
Albumin	3.17^c^	3.57^a^	3.56^a^	3.39^b^	0.02	<0.01	<0.01	0.04
Globulin	3.46^a^	3.30^a^	3.02^b^	3.47^a^	0.03	0.01	<0.01	0.03
BUN	13.48^a^	10.97^c^	11.89^b^	11.39^bc^	0.11	<0.01	<0.01	0.26

Control group = basal diet consists of German grass and concentrate with 14.9% CP and 11.0 MJ ME/kg DM without herbs; Plantain group = basal diet + 100 g shade-dried plantain powder/cow per day; Lemongrass group = basal diet + 100 g shade-dried lemongrass powder/cow per day; Combined group = basal diet + 50 g shade-dried plantain and 50 g shade-dried lemongrass powder/cow per day; SEM = standard error of mean.

*Probability of treatment effects: D = effects of experimental diets; T = effect of time; D × T = interaction between diets and time; ^a–c^Means within a row, denoted by distinct superscripts indicate significant differences (p ≤ 0.05) for the experimental diets.

mg/dl = milligram per decilter; TC = total cholesterol; HDL-C = high-density lipoprotein cholesterol; LDL-C = low-density lipoprotein cholesterol; VLDL-C = very low-density lipoprotein cholesterol; BUN = blood urea nitrogen.

### 3.4. Serum and milk antioxidants

Herbal supplementation caused a similar improvement in the serum and milk antioxidant status, as assessed through the TAC and catalase contents (**p** *<* 0.01; Table 5). Furthermore, compared with the control group, herbal supplementation resulted in greater serum (7–8%) and milk (15–23%) SOD and higher serum (5–7%) and milk (8–12%) GPx concentrations (**p** < 0.01). Compared with the control group, the lemongrass or combined group resulted in around 27% and 19% more β-carotene in milk, respectively (**p** < 0.01).

**Table 5:** Effects of herbal supplementation on serum and milk antioxidant contents in tropical crossbred cows.

Variables	Control	Herbs-supplemented groups			SEM	p-value*		
		Plantain	Lemongrass	Combined		D	T	D×T
**Serum antioxidants**								
TAC (mg/l)	191.23^c^	200.55^a^	198.16^b^	198.61^b^	0.27	<0.01	<0.01	0.01
SOD (%)	97.59^b^	105.03^a^	104.97^a^	104.26^a^	0.20	<0.01	0.01	0.92
GPx (mU/ml)	218.19^c^	233.83^a^	228.48^b^	231.87^ab^	0.59	<0.01	<0.01	0.08
Catalase (mU/ml)	6.73^c^	8.20^a^	7.94^ab^	7.75^b^	0.07	<0.01	0.03	0.48
**Milk antioxidants**								
DPPH (%)	19.83^b^	21.83^a^	22.03^a^	21.89^a^	0.08	<0.01	0.50	0.95
TAC (mg/l)	32.32^c^	36.36^a^	35.23^b^	35.70^ab^	0.14	<0.01	<0.01	0.37
SOD (%)	18.33^c^	22.60^a^	21.11^b^	21.32^b^	0.09	<0.01	<0.01	0.01
GPx (mU/ml)	29.99^d^	32.48^c^	33.19^b^	33.67^a^	0.07	<0.01	0.06	0.34
Catalase (mU/ml)	2.01^b^	2.11^a^	2.11^a^	2.10^a^	0.01	<0.01	<0.01	0.01
β-carotene (mg/kg)	0.26^c^	0.27^c^	0.33^a^	0.31^b^	0.01	<0.01	<0.01	0.30

Control group = basal diet consists of German grass and concentrate with 14.9% CP and 11.0 MJ ME/kg DM without herbs; Plantain group = basal diet + 100 g shade-dried plantain powder/cow per day; Lemongrass group = basal diet + 100 g shade-dried lemongrass powder/cow per day; Combined group = basal diet + 50 g shade-dried plantain and 50 g shade-dried lemongrass powder/cow per day; SEM = standard error of mean.

*Probability of treatment effects: D = effects of experimental diets; T = effect of time; D × T = interaction between experimental diets and time; ^a–d^Means within a row, denoted by distinct superscripts indicate significant differences (p ≤ 0.05) for the experimental diets.

mg/l = milligram per liter; % = percentage; mU/ml = milliunit per milliliter; mg/kg = milligram per kilogram; TAC = total antioxidants capacity; SOD = superoxide dismutase; GPx = glutathione peroxidase; DPPH = 2, 2‒diphenyl‒1‒picryl hydroxyl.

### 3.5. Milk fatty acid profiles

Compared with the control group, herbal supplementation resulted in 4–7% lower saturated fatty acids (SFAs) and 6‒10% higher unsaturated fatty acids (USFAs) in milk with better monounsaturated fatty acids (MUFAs) (p *≤* 0.01; Table 6). Compared with those in the control group, the PUFAs content in the lemongrass group was 1.41-fold greater (p *=* 0.09). Furthermore, herbal supplementation increased the amount of n-3 fatty acids, with 2.89- and 3.56-fold greater fatty acids in milk cows given the lemongrass or combination of plantain-lemongrass, respectively (p < 0.01). In contrast, milk n-6 fatty acid content was unchanged among all the groups (p *=* 0.31). Additionally, the fatty acid profiles changed substantially in response to all groups except C12:0, C14:0, C18:1, and C20:1 fatty acids. Compared with those in the control group, the C16:0 and C16:1 in the herbal supplemented groups were lower and greater, respectively (p ≤ 0.01).

**Table 6:** Effects of herbal supplementation on milk fatty acid profiles (g/100 g fatty acid) in tropical crossbred cows.

Variables	Control (n = 10)	Herbs-supplemented groups			SEM	p-value
		Plantain (n = 10)	Lemongrass (n = 10)	Combined (n = 08)		
C4:0	2.55^a^±0.03	2.24^b^±0.09	2.41^ab^±0.08	2.29^b^±0.09	0.04	0.04
C12:0	0.35±0.02	0.34±0.02	0.26±0.03	0.29±0.02	0.01	0.06
C14:0	8.30±0.37	8.46±0.22	8.16±0.34	8.96±0.43	0.18	0.45
C15:0	0.35^c^±0.02	1.30^b^±0.04	1.64^a^±0.04	1.73^a^±0.06	0.14	<0.01
C16:0	39.26^a^±1.10	36.53^ab^±0.50	34.83^b^±0.43	34.43^b^±0.71	0.59	0.01
C18:0	6.74^a^±0.19	6.27^ab^±0.11	5.42^c^±0.05	5.79^bc^±0.21	0.14	<0.01
C20:0	0.24^b^±0.12	0.16^b^±0.10	0.67^a^±0.05	0.71^a^±0.03	0.07	0.01
C22:0	-*	-*	0.09^a^±0.01	0.11^a^±0.01	0.01	<0.01
C14:1	2.39^b^±0.07	2.20^b^±0.04	3.07^a^±0.04	3.18^a^±0.10	0.11	<0.01
C16:1	1.40^c^±0.05	3.28^a^±0.06	1.82^b^±0.07	2.05^b^±0.05	0.18	<0.01
C18:1	33.99±0.46	34.05±0.29	34.97±0.22	34.70±0.38	0.20	0.27
C20:1	0.05±0.04	0.08±0.04	-*	0.14±0.04	0.02	0.13
C18:3 (n-3)	0.36^c^±0.03	0.53^c^±0.02	1.04^b^±0.08	1.28^a^±0.11	0.09	<0.01
C18:2 (n-6)	4.42±0.39	4.55±0.54	5.62±0.57	4.48±0.40	0.24	0.31
SFAs	57.80^a^±0.74	55.30^b^±0.33	53.48^b^±0.46	54.31^b^±0.43	0.46	<0.01
USFAs	42.20^b^±0.74	44.70^a^±0.33	46.52^a^±0.46	45.69^a^±0.43	0.46	<0.01
MUFAs	37.83^a^±0.37	39.61^b^±0.27	39.87^b^±0.27	40.07^b^±0.52	0.29	0.01
PUFAs	4.78±0.39	5.07±0.55	6.66±0.51	5.76±0.43	0.27	0.09

Control group = basal diet consists of German grass and concentrate with 14.9% CP and 11.0 MJ ME/kg DM without herbs; Plantain group = basal diet + 100 g shade-dried plantain powder/cow per day; Lemongrass group = basal diet + 100 g shade-dried lemongrass powder/cow per day; Combined group = basal diet + 50 g shade-dried plantain and 50 g shade-dried lemongrass powder/cow per day; SEM = standard error of mean.

^a–c^Means within a row, denoted by distinct superscripts, indicates significant (p ≤ 0.05).

SFAs = saturated fatty acids (C4:0 + C12:0 + C14:0 + C15:0 + C16:0 + C18:0 + C20:0 + C22:0); USFAs = unsaturated fatty acids (C14:1 + C16:1 + C18:1 + C18:2 + C18:3 + C20:1); MUFAs = monounsaturated fatty acids (C14:1 + C16:1 + C18:1 + C20:1); PUFAs = polyunsaturated fatty acids (C18:2 + C18:3); *- = not detected.

### 3.6. Cost‒benefit analysis

**Fig 1:**
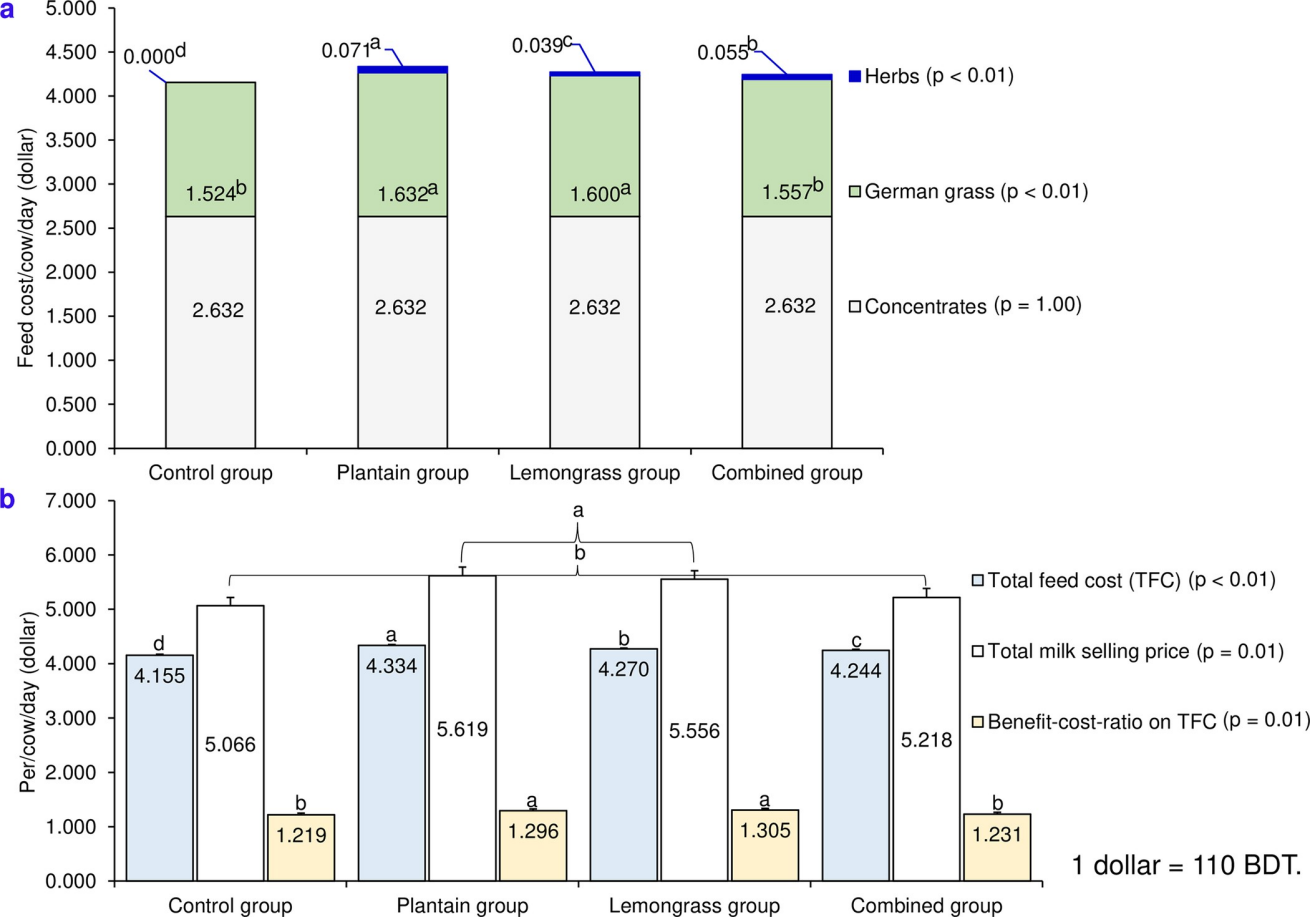
Effects of experimental diets^**1**^ on feed ingredients (a), total feed cost (TFC), total milk selling price, and benefit–cost–ratio (BCR) (b) in tropical crossbred cows. ^**1**^Experimental diets: Control group = basal diet consists of German grass and concentrate with 14.9% CP and 11.0 MJ ME/kg DM without herbs; Plantain group = basal diet + 100 g shade-dried plantain powder/cow per day; Lemongrass group = basal diet + 100 g shade-dried lemongrass powder/cow per day; Combined group = basal diet + 50 g shade-dried plantain and 50 g shade-dried lemongrass powder/cow per day; ^**a–d**^Means within a row, denoted by distinct superscripts indicate significant differences (p ≤ 0.05). BDT = Bangladeshi taka.

The costs of feed ingredients, excluding concentrate, varied significantly among all dietary groups (Fig 1A; p < 0.01). Compared with the control group, the plantain, combined, and lemongrass groups presented greater herb costs, with significant differences among these groups (p < 0.01). Additionally, compared with the control and combined groups (p > 0.05), the plantain or lemongrass group presented significantly greater costs for German grass (Fig 1A). The total feed cost (TFC) and milk selling price differed substantially among all dietary groups, with the lowest TFC and total milk selling price observed in the control group and the optimum TFC and total milk selling price found in the plantain group (Fig 1B). Compared with the control (1.22) and combined (1.23) groups (p > 0.05), significantly greater BCR values were detected in the lemongrass (1.31) and plantain (1.30) groups, which were comparable (p > 0.05).

## 4. Discussion

This study is unique in that it aimed to assess whether shade-dried plantain, lemongrass, or their combined antioxidants (phytochemicals) may improve serum and milk antioxidants in cows. Both shade-dried herbs are rich in antioxidants because they have more significant amounts of DPPH (27.43% and 15.27%), total phenolics (24.67 mg GAE dry sample and 19.83 mg GAE/g dry sample), and total flavonoids (5.29 mg QE/g dry sample and 10.51 mg QE/g dry sample; Table 1). The double bonds in DPPH, gallic acid, and quercetin act as potent antioxidants that scavenge free radicals and may increase blood and milk antioxidants in dairy cows [29,30]. Furthermore, a lower amount of gallic acid effectively scavenges detrimental superoxide and hydroxyl radicals. It increases immunity by limiting the production of cytokineand histamine, which may lead to greater antioxidants in the blood and milk of dairy cows [31]. In addition to these ample phytochemicals with greater USFAs, PUFAs, and n-3 fatty acids (Table 1) in plantain, lemongrass, or mixture of plantain-lemongrass may improve the corresponding fatty acids in the milk of cows [32].

### 4.1. Production performance

Feeding 100 g of lemongrass powder daily to beef cattle [4] and plantain with diverse pastures to dairy cows [6] have no effect on DM consumption, which contradicts current research. In addition, supplementing 10 g DM of plantain to sheep [9,13] and around 4 g DM of lemongrass to Burki goats [33] improve DM consumption, supporting the current findings. These better results in the plantain or lemongrass group might be attributed to their antioxidant, hepatoprotective, and anti-inflammatory activities, which trigger the release of salivary, gastric and pancreatic enzymes, especially pancreatic lipase and amylase [34]. Phytochemicals in plantain or lemongrass have antimicrobial activity, which may improve DM consumption in both groups [16,35]. For example, phytochemicals acteoside in plantain and citral in lemongrass nourish the microbiome and enhance cellulolytic and amylolytic bacteria, thus resulting in DM consumption [4,10,36]. However, the combined group failed to improve DM intake in the current study, similar to lambs given lemongrass-roselle supplementation [15]. These results might be attributed to the action of mixed phytochemicals of plantain-lemongrass, which may negatively affect the rumen microbiome, thus resulting in low DM consumption compared with that of the plantain or lemongrass group [15].

Dairy cows given plantain or lemongrass presented a 10–11% greater milk yield, whereas earlier studies revealed that cows grazed swards or forbs with 18.4% or 23.0% plantain on a DM basis or fed dairy goats or ewes 5–10 g of lemongrass powder daily improve milk yield by 7–27% and enhance ECM, FCM, TS, SNF, lactose, protein, and fat. Furthermore, cows supplemented with herbs produce milk with different yields and compositions [6,37]. Cows given 350 g of pomegranate seeds [37] or 100 g of a traditional Chinese herbal mixture of Indian mint, banjiryun, chenpi, or gan-cao [38] do not affect milk yield.

The greater milk yield in the plantain or lemongrass group might be attributed to better serum antioxidant levels, albumin concentrations, DM intake, and nutrient digestibility in dairy cows. Greater amounts of serum antioxidants scavenge free radicals, increasing immunity, DM intake, and nutritional digestibility in dairy cows [39,40]. Elevated serum albumin concentrations may have contributed to the production response [41], whereas greater DM intake and better nutritional digestion and absorption might have accelerated milk yield in both the plantain and lemongrass [14]. However, the combined group failed to improve milk yield due to lower DM intake and nutrient digestibility than the plantain or lemongrass group did [42]. Moreover, the combined group presented better serum antioxidant, albumin, and lower BUN levels than did the control group. This could account for the combined group showing a numerically higher milk yield than the control, although the difference was not statistically significant.

Plantain and lemongrass phytochemicals stimulate rumen propionic acid-producing bacteria, increasing rumen propionate [14,43]. This propionate is a vital precursor for the synthesis of milk lactose, improving the lactose yield in both the plantain and lemongrass. The lower serum BUN may have resulted from better mammary gland amino acids partitioning for milk protein biosynthesis. Herb phytochemicals and zinc are transported to milk from the diet via blood, then bound to protein, increasing milk protein yield, and alleviating oxidative stress [44]. The improved CF, NDF, and ADF digestion in the lemongrass or plantain group might have produced higher acetate levels, thus resulting in increased milk fat yield [14]. Compared with the control group, the combined group tended to produce more milk lactose, protein, and fat, probably due to greater nutrient digestion and absorption.

### 4.2. Apparent nutrient digestibility

Compared with the control group, the plantain or lemongrass group substantially improved nutrient digestibility, with the exception of CP, but not the combined group, which supported previous findings [13,15,16]. Plantain, as a species of pasture, improves nutrient utilization in dairy cows [45], while feeding sheep 10 g of DM of plantain daily increases the digestibility of all nutrients except CP [13], supporting the current findings. Besides, the daily addition of 100 g of lemongrass powder to beef [4,46] and 5 or 10 g of lemongrass powder to ewes [16] improve the DM, OM, EE, NDF, and ADF digestibility compared with those of the respective control groups, which aligns with the results of the present study. This better nutrient digestibility in the plantain or lemongrass group might be attributed to greater feed quality, such as phytochemicals in plantain or lemongrass, which may improve nutrient digestibility in both groups [16,35]. For example, the phytochemicals acteoside in plantain and citral in lemongrass enhance the beneficial rumen microbiome, resulting in increased rumen fermentation, feed intake, microbial protein supply, and nutrient utilization [10,36]. Furthermore, plantain herb suppresses parasites [9] and increases nutrient turnover [35], whereas lemongrass modifies the rumen microbial population, especially protozoa [4,15], which may improve nutrient digestibility. The plantain or lemongrass group also had greater DM intake, which may improve gut feed retention and nutrient digestibility. Additionally, similar to how the diverse phytochemicals in lemongrass and roselle have no effect on the rumen protozoal count in ewes [15], the phytochemicals in the plantain-lemongrass mixture might also play a role, as the nutritional digestibility of the combined group remained unchanged. However, the plantain group presented 4.48% lower CP digestibility than did the control group since aucubin may reduce the abundance of hyperammonia-producing bacteria, lowering the rumen ammonia concentration and protein digestibility [47].

### 4.3. Serum metabolites and protein indices

Compared with the control group, the herbal supplementation groups presented greater serum HDL-C and lower triglyceride, TC, LDL-C, and VLDL-C concentrations, supporting previous findings [13,37]. These beneficial modifications may be caused by exogenous (diet) or endogenous (body) mechanisms [48]. The use of plantain as a pasture component in dairy cows [49] and as a supplement to sheep [9] has been inconsistent, whereas the use of acteoside as an additive in ewes [48] has been shown to positively affect the levels of serum metabolites, supporting the findings of the present study. Additionally, lemongrass supplementation reduces serum cholesterol in ewes [17] but not in steers [17], whereas lemon oil or tea improves beneficial serum lipid metabolites in both mice and rats [50]. This beneficial change might be attributed to the greater amount of MUFAs and PUFAs in both herbs and their ability to transfer from the gastrointestinal tract to the bloodstream. These MUFAs and PUFAs in the blood directly increase HDL-C and reduce triglyceride, TC, and VLDL-C levels [51]. Additionally, phytochemicals (saponins, alkaloids, flavonoids, and tannins) in both herbs might be reduced the biohydrogenation process in the rumen, which resulting in higher USFAs flow in the blood, lower serum triglyceride, and increase HDL-C in dairy cows [32]. Both herbal phytochemicals (quercetin and saponins) have antihypercholesterolemic activity, which reduces cholesterol and triglyceride synthesis and increases their fecal flow [52]. In addition, both herbs’ antioxidant and anti-inflammatory properties stimulate lipoprotein lipase and lecithin cholesterol acetyltransferase enzyme secretion, which breaks down triglycerides into free fatty acids and glycerol, lowering VLDL-C and triglyceride [53].

Herbal supplements reduce BUN in dairy cows via the presence of saponins in herbs, which suppress hyperammonia-producing bacteria such as *Ruminobacter amylophilus*, *Prevotella* spp., and proteolytic bacteria. Tannins in both herbs bind with dietary protein, preventing ruminal breakdown and lowering rumen ammonia and BUN levels in dairy cows [6,7]. Plantain had the lowest amount of BUN. This may be due to aucubin in plantain lowering the amount of ammonia-N in the rumen [10]. In addition, plantain and lemongrass given to ruminants have inconsistent serum total protein concentrations [7,16,17]. In the present study, the anti-inflammatory effects of both herbs may increase the serum albumin concentration and decrease the globulin concentration in dairy cows [41].

### 4.4. Serum and milk antioxidant contents

**Fig 2:**
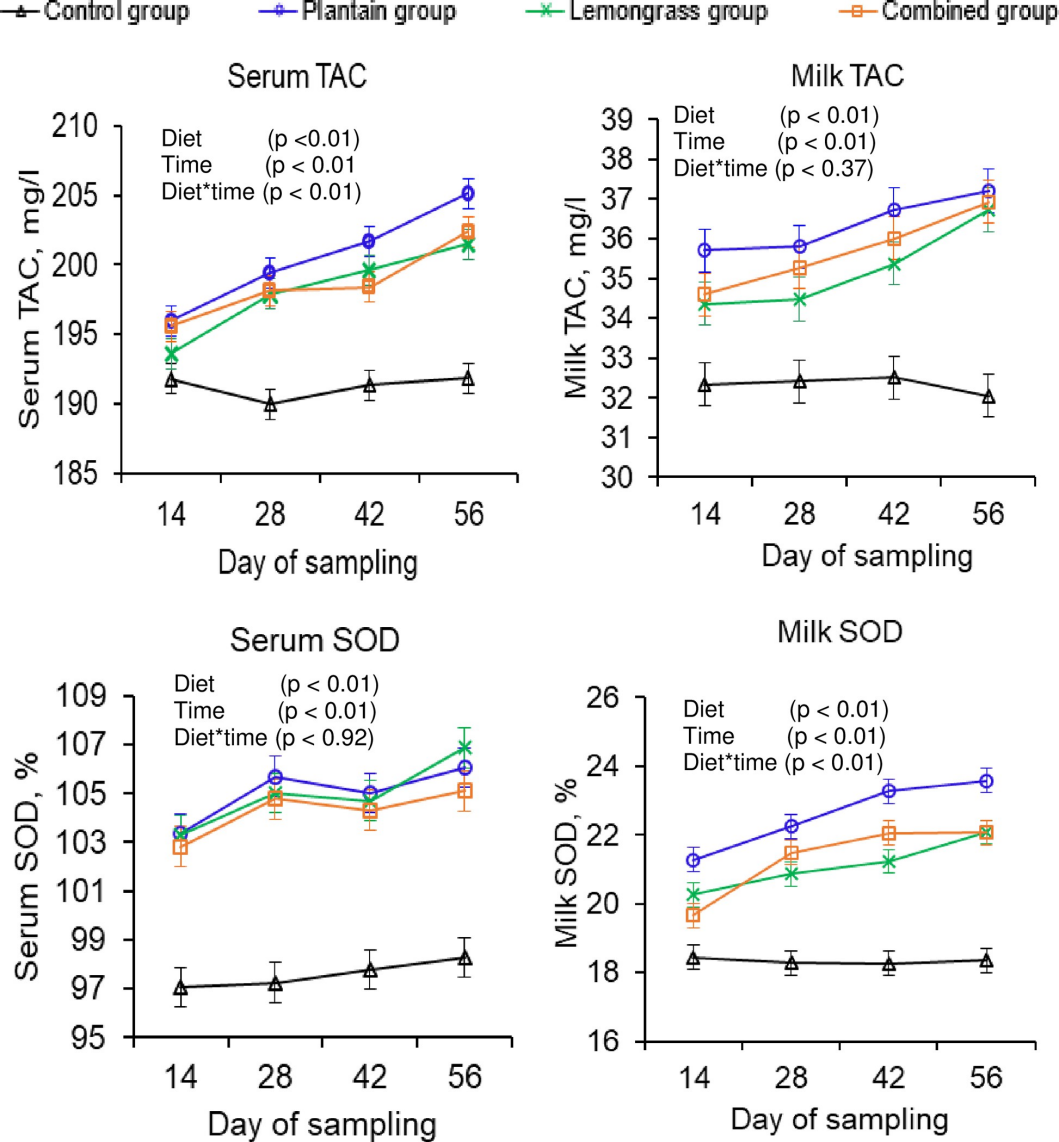
Effects of experimental diets^1^ on total antioxidant capacity (TAC) and superoxide dismutase (SOD) concentrations in serum and milk of tropical crossbred cows.

**Fig 3:**
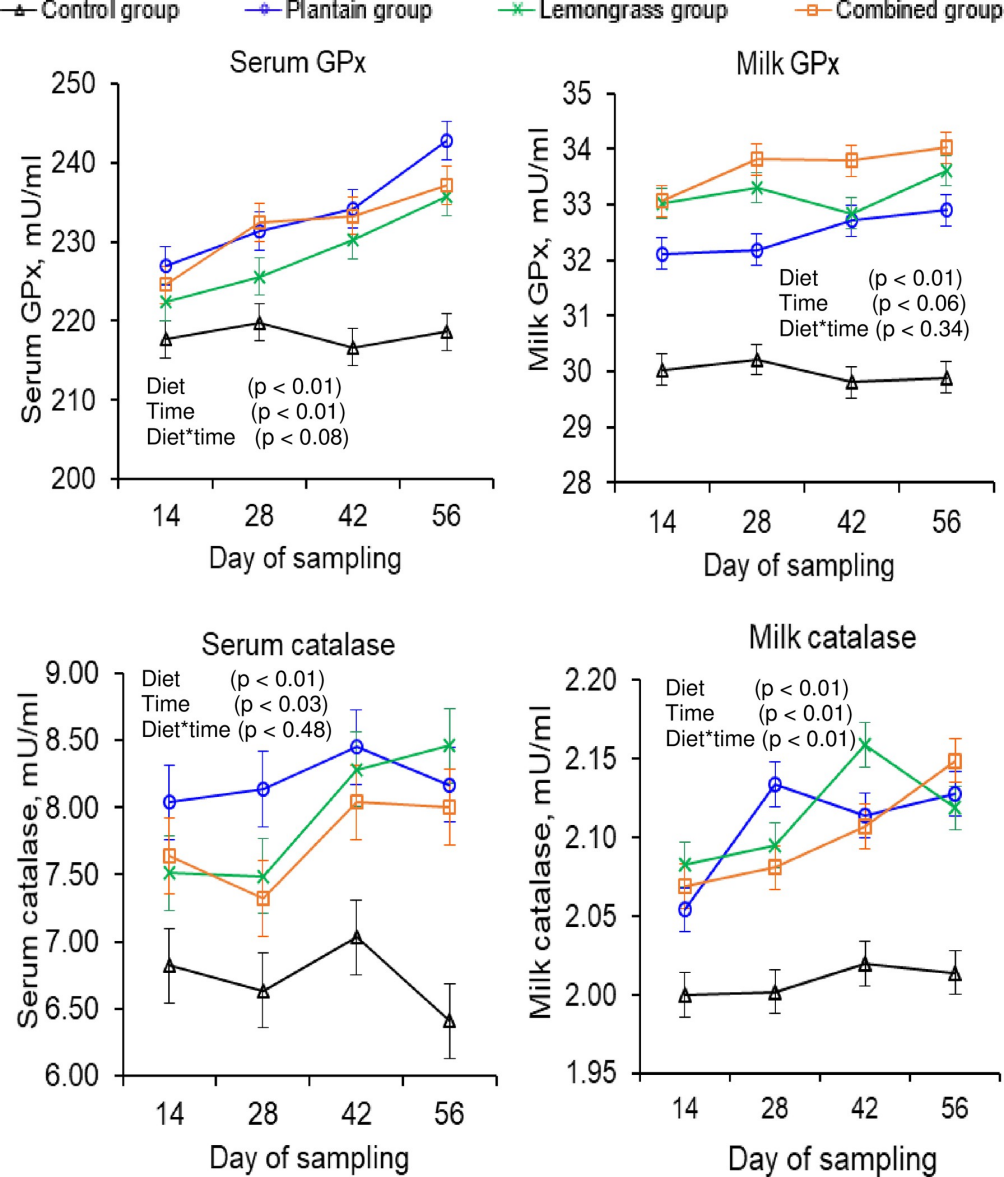
Effects of experimental diets^1^ on glutathione peroxidase (GPx) and catalase concentrations in serum and milk of tropical crossbred cows. ^1^Experimental diets: Control group = basal diet consists of German grass and concentrate with 14.9% CP and 11.0 MJ ME/kg DM without herbs; Plantain group = basal diet + 100 g shade-dried plantain powder/cow per day; Lemongrass group = basal diet + 100 g shade-dried lemongrass powder/cow per day; Combined group = basal diet + 50 g shade-dried plantain and 50 g shade-dried lemongrass powder/cow per day; p ≤ 0.05 indicates significant; mg/l = milligram per liter; mU/ml = milliunit per milliliter.

The concentration of enzymatic antioxidants in dairy cows is modulated by physiological phenomena, environmental temperature, time, and days of milking [54]. Consequently, serum and milk antioxidant enzymes such as SOD, GPx, catalase, and TAC were inconsistent in dairy cows at various sampling times, but compared with the control group, herbal supplementation improved these antioxidant concentrations in the blood and milk, supporting previous findings [37]. Furthermore, dairy animals are fed phytochemicals through grape seeds and subsequently transferred to blood (+10%) or milk (+32%) [30]. As previously reported [30,37], herbal supplementation substantially improved serum (4–8%) and milk (8–23%) antioxidant levels, which increased with increasing days of feeding (*Figs 2 and 3*). Similarly, dairy cows given terpene via pasture and phenolic acid via rosemary leaves show improved blood and milk phytochemicals concentrations and increased oxidative stability [20,39]. Plantain and lemongrass as supplements improve the serum SOD, GPx, and catalase concentrations in sheep [13] and dairy cows [55], respectively. However, dairy cows given an extra 7.8 g of total extractable phenols of eucalyptus, thyme, and anise essential oils or buffalo fed 0.75–2.25 g of flavonoids via mulberry leaves daily unable to improve the serum and milk antioxidant concentrations [56,57]. This variation may be due to an inappropriate dose, nature, type of phytochemicals, or heat stress [56,57]. In addition, the permeability of these phytochemicals from herbs to milk is another important factor. Some phytochemicals may pass through blood to milk, but some cannot [20]. For example, these phytochemicals (caffeic acid, p-coumaric acid, luteolin, quercetin, and gallic acid) in both herbs can be transferred from herbs to milk via blood, increasing milk TAC levels, which supports previous research [20,58]. In the present study, dairy cows given an average of 3.0 g of phenolic (gallic acid) or flavonoid (quercetin) via plantain or lemongrass daily improved the blood and milk antioxidants SOD, GPx, catalase, and TAC, which is consistent with previous findings [30,37]. These flavonoids and phenolics in both herbs inhibit intracellular oxidative damage by donating hydrogen ions to reduce free radical activity, improving serum and milk antioxidants [29,42]. Furthermore, in the present study, cows receiving herbal supplements exhibit higher serum albumin concentrations, i.e., better Cys34 residues, which neutralize free radicals such as reactive oxygen and nitrogen species, hydrogen peroxide, hypochlorous acid, and superoxide anions, improving serum antioxidants [59]. Moreover, these elevated serum antioxidants could improve milk SOD, GPx, catalase, and TAC [58]. The antioxidant citral in lemongrass, which aids in vitamin-A synthesis in the liver, may contribute to higher milk β-carotene levels in the lemongrass group [4].

### 4.5. Milk fatty acid profiles

Breed, lactation stage, mastitis, diet, and environmental conditions strongly influence the fatty acid profiles of milk [14]. Herbal supplementation substantially reduced SFAs and improved USFAs, MUFAs, and n-3 fatty acids in cow milk, supporting previous findings [7,14]. These beneficial modifications of fatty acids may be due to the rich USFAs, MUFAs, PUFAs, and phytochemical profiles of plantain or lemongrass. Usually, biohydrogenation occurs in the rumen when bacteria hydrogenate USFAs and PUFAs, especially n-3 and n-6 fatty acids, from feed digestion in the rumen, increasing milk SFAs. However, phytochemicals in plantain (acteoside and tannins) and lemongrass (hexadecanoic and octadecadienoic) reduce the abundance of rumen microbes responsible for biohydrogenation in dairy cows [32]. This improved USFAs, n-3, and n-6 fatty acids concentrations in the rumen, which are absorbed from the small intestine and then transferred from the blood to the milk. In addition, limonene and terpenes in lemongrass improved milk PUFAs and n-3 fatty acids levels [60]. Herbal supplementation elevated β-carotene and antioxidant capacity in milk in the present study, which may have reduced free radical production and oxidative PUFAs degradation and improved the levels of MUFAs and PUFAs, especially n-3 fatty acids in milk [61]. Furthermore, the elevated serum albumin levels in the herbal supplemented groups may be responsible for the greater PUFAs levels since albumin binds to PUFAs and acts as an antioxidant to prevent oxidation [59]. Additionally, the herbal supplementation groups presented more C14:1 and C16:1 fatty acids owing to greater stearoyl-CoA desaturase (SCD), which converts long-chain SFAs to MUFAs in the mammary glands of dairy cows [51]. These beneficial changes might be attributed to the greater antioxidant capacity of herbal supplements, which donates H ions to convert SFAs to the corresponding MUFAs in the mammary glands through the SCD enzyme [51]. Moreover, herbal supplementation reduced the C4, C16, and C18 fatty acid contents in milk resulting from lower total serum cholesterol [62]. Additionally, plantain and lemongrass contain greater percentages of USFAs (79.60% and 74.33%), MUFAs (92.88% and 87.52%), PUFAs (7.12% and 12.48%), and n-3 fatty acids (46.07% and 57.89%), which might improve the corresponding milk fatty acids in dairy cows [14,16].

### 4.6. Cost‒benefit analysis

Consistent with the results of the present study, Holstein-tropical crossbred cows in this region usually offer greater amounts of concentrate feed to fulfil their nutritional requirements due to the unavailability of leguminous fodders [63]. Previous findings [64,65] have shown that concentrate account for the major cost of dairy farming in this region, which supports the current study. Moreover, the cost of the concentrate was similar among all dietary groups in this study. However, the TFC was greater in the herbal supplemented groups because of the supplementation of herbs and higher intake of grass. Compared with those in the control and combined groups, better BCR were obtained in the lemongrass or plantain group because of the higher milk selling price related to TFC. Compared with the control group, the combined group presented a greater TFC and similar BCR due to the use of herbs and a higher milk selling price, respectively.

## 5. Conclusions

Cows fed shade-dried herb either plantain or lemongrass individually presented better dry matter intake and nutrient digestibility, resulting in a 10–11% greater milk yield than the control. Compared with those in the control group, the milk antioxidant concentrations in the plantain, lemongrass, and combined groups improved by 12.27%, 9.95%, and 10.88%, respectively. In addition, the plantain, lemongrass, and combined groups improved the levels of beneficial serum metabolites, milk unsaturated fatty acids, notably n-3, and the combined group presented the optimum n-3 fatty acid contents than did the control group. Compared with those of the control and combined groups, almost 7.0% and 6.0% greater benefit-cost-ratio on a total feed cost basis were obtained for the lemongrass and plantain groups, respectively. Finally it is concluded that, shade-dried herbs, including plantain, lemongrass, or their combination, individually enhanced the levels of beneficial serum metabolites, serum and milk antioxidants, and unsaturated fatty acids. In addition, shade-dried plantain or lemongrass improved dry matter intake, nutrient digestibility, milk yield, and overall farm profitability.
